# Rapid Point-Of-Care Serology and Clinical History Assessment Increase Protection Provided by RT-PCR Screening: A Pilot Study Involving Three Nursing Homes in Brescia, a Hotspot of Lombardy

**DOI:** 10.3389/fpubh.2021.649524

**Published:** 2021-06-24

**Authors:** Antonella Savio, Stefano Calza, Gianbattista Guerrini, Valentina Romano, Eleonora Marchina

**Affiliations:** ^1^Histopathology Department, The Royal Marsden Hospital, London, United Kingdom; ^2^Department of Molecular and Translational Medicine, University of Brescia, Brescia, Italy; ^3^Nursing Homes Villa Elisa and Arici-Sega, Brescia, Italy; ^4^Nursing Home Casa Industria, Brescia, Italy

**Keywords:** COVID-19, SARS–CoV−2, asymptomatic transmission, nursing home, on-site testing, COVID-19 symptoms, RT-PCR screening, rapid serological assays

## Abstract

**Background:** COVID-19 outbursts have been registered worldwide within care homes with asymptomatic transmission combined with shortage/inaccuracy of diagnostic tests undermining the efforts at containment of the disease. Nursing facilities in Lombardy (Italy) were left with no, or limited, access to testing for 8 weeks after the outbreak of COVID-19.

**Methods:** This study includes 246 residents and 286 workers of three different nursing homes in Brescia-Lombardy. Clinical questionnaires and rapid serology tests were devised to integrate the data of the first available RT-PCR screening. Follow-up serology after 60-days was performed on 67 of 86 workers with positive serology or clinically suspicious.

**Findings:** Thirty-seven residents and 18 workers had previous positive RT-PCR. Thorough screening disclosed two additional RT-PCR-positive workers. Serology screening revealed antibodies in 59 residents and 48 workers, including 32/37 residents and all workers previously positive at RT-PCR. Follow up serology disclosed antibodies in two additional workers with recent symptoms at the time of screening. The professionals in close contact with residents had more infections (47/226–20.79% vs. 1/60–1.66%; *p* = 0.00013 Fisher exact-test). A suspicious clinical score was present in 44/64 residents and in 41/50 workers who tested positive with either method with totally asymptomatic disease more frequent among residents 28.1 vs. 10.0% (*p* = 0.019 Fisher exact-test).

**Interpretation:** Based on the available RT-PCR ± results at the time of symptoms/contacts, our integrated clinical and serological screening demonstrated sensitivity 89% and specificity 87%. This multimodal assessment proved extremely useful in understanding the viral spread in nursing homes, in defining its stage and in implementing protective measures. Rapid serology tests demonstrated efficient and particularly suited for older people less able to move/cooperate.

## Introduction

Older adults with or without serious underlying medical conditions are at high risk for severe forms of COVID-19 (1, 2). Old people cared for in nursing homes frequently bear co-morbidities, are highly dependent on assistance and continuous close contact by multiple carers and are frequently hosted in shared rooms. In these circumstances the occurrence of severe outbreaks is easily predictable (3, 4).

Italy was the first country in Europe to be hit by SARS-Cov2, and Lombardy the first and most affected region, especially the provinces of Brescia and Bergamo (5).

Specific behavioral restrictions, including lockdown and social distancing, have been enacted in care homes in Brescia from February 28th, a week after the beginning of the COVID-19 outbreak in Lombardy and well in advance of European Community guidelines released in March. Nevertheless, outbursts of infection in many nursing homes were registered.

In Lombardy, the impact of COVID-19 epidemics was so overwhelming that no tests were available for several weeks except for those patients presenting with severe symptoms to Accident and Emergency (6).

In this chaotic emergency situation, care homes were laced at “the back of the queue.” Virology swabs were available for symptomatic residents and healthcare workers (HCW) only from April 4th and a staff-only RT-PCR screening was undertaken at the end of April.

Given the shortage of any other laboratory tests, rapid serology tests associated with recording of clinical data appeared a promising integration of virology screening in identifying false negative RT-PCR cases (7) and asymptomatic infections and in tailoring additional protective measures.

## Materials and Methods

The cohort of this study included residents and staff of three differently sized care homes in Brescia (Italy) managed by the same medical direction during the COVID-19 outbreak: “CI,” a long-established geriatric care home for up to 130 residents in central historic buildings, “VE” and “AS,” hosting up to 62 and 120 residents in modern buildings on the outskirts of town. The number of single rooms was, respectively, 4 (CI), 30 (VE), and 48 (AS). Rooms occupied by three or more residents were present in the historic central building only (CI). The smallest nursing home (VE) had no registered COVID-related deaths amongst residents at the time of our screening, while several cases had been already registered in both other residences.

The research involved 532 persons including 246 residents (age range 27–100; mean 83; males 28%) and 286 regular and shift HCW (age range 23–70 years; mean 46; males 14%) (Tables 1, 2). The adherence to the study was voluntary: 13 residents and 7 HCW refused participation. A single HCW was excluded because she was quarantined at home. Testing was non-practicable for one resident.

**Table 1:** Data of 246 residents of three nursing homes (CI, VE, AS).

**Residents**	**CI**	**VE**	**AS**	**Total**
Total	107	51	88	246
Males	29	11	30	70
Females	78	40	58	176
<70 y/o	6	3	12	21
70–79 y/o	14	13	19	46
80–89 y/o	38	19	30	87
90–99 y/o	49	16	17	82
>99 y/o	0	0	1	1
Age range	42–99	66–97	27–100	27–100
Mean age	85	84	80	83
RT-PCR positive^*^	11 (10%)	3 (6%)	25 (28%)	39 (16%)
Serology positive	23 (21%)	7 (14%)	29 (33%)	59 (24%)
Clinically suspicious (score 2 and above)	22 (21%)	8 (16%)	47 (53%)	77 (31%)
RT-PCR and/or serology positive	23 (21%)	8 (16%)	32 (36%)	64 (26%)

* *With RT-PCR positive test preceding clinico-serological screening and/or following detection of positive serology*.

**Table 2:** Data of 286 staff of three nursing homes (CI, VE, AS).

**HCW (246 F 40M mean age 46)**	**Total**	RT-PCR positive^*^	Serology screening positive ^*^^#^^∧^	**Clinical score ≥2**
Doctors	10	2 (20%)	3 (30%)	5 (50%)
Nurses	39	6 (15%)	12 (31%)	14 (36%)
Health care assistants OSS	80	6 (7%)	19 (24%)	40 (50%)
Social welfare assistants ASA	83	5 (6%)	14 (17%)	30 (36%)
Physiotherapists	10	1 (10%)	1 (10%)	3 (30%)
Educators/psychologists	13	0	0	2 (15%)
Catering staff	16	0	0	1 (6%)
Maintenance workers	3	0	0	1 (33%)
Administrative employees	16	0	1 (6%)	4 (25%)
Cleaners/laundry workers	12	0	0	1 (8%)
Beautician/hairdresser	4	0	0	1 (25%)
TOT multiple employment	51	5 (10%)	10 (20%)	16 (31%)
TOT sole employment	235	15 (6%)	40 (17%)	86 (30%)
TOTAL HCW	286	20 (7%)	50 (17.5%)	102 (36%)

* *Includes all workers with positive test: preceding the thorough triple screening and/or at the time of screening and/or after the screening*.

# *Total 50 cases: 44 HCW positive to serology screening plus 4 HCW with discrepant weakly positive at re-testing after 1 week only plus 2 cases positive at re-testing after 60 days*.

∧ *Total serology positive corresponds to all 50 cases with either test positive as no RT-PCR positive cases with negative serology in this group*.

The residents included 184 people with dementia, 58 of which had Alzheimer's or other dementia with wandering, and 20 in persistent vegetative state, including all six residents aged <65 (age range 23–61).

Regular HCW included professionals in close contact with residents (doctors, nurses, healthcare assistants, social health workers, physiotherapists, hairdressers, and beauticians) plus cleaners, catering staff, administrative workers. Shift workers were represented by night shift doctors and nurses and amounted to 51 workers.

Integrated triple screening of HCW was performed between April 23 and May 14, 2020.

Double clinico-serological screening of residents was performed May 19–June 30, 2020.

Data analysis was undertaken between May and July 2020.

### Clinical Assessment

The clinical questionnaire devised for all HCW included a list of 33 symptoms linked to COVID-19, exposure to proven infected persons, precautional isolation measures, results of RT-PCR tests, data about underlying health conditions and routine or recent treatments. The list of symptoms (Supplementary Table 1) was drawn from the existing literature on March–April 2020 (1, 8–11) or inferred from the reports of the self-help Facebook “Coronavirus, CoV-2 and COVID-19 group for doctors only” created at the beginning of the European outburst and joined by more than 100.000 Italian and European doctors. A simplified list comprising the 18 more objectifiable symptoms (Supplementary Table 1) was completed by their doctors for all residents considered less able to report their symptoms. Symptoms referring to the period of SARS-Cov2 diffusion in Italy only were recorded, starting in February 2020. Exclusion of usual symptoms related to chronic conditions or to allergy was asked.

The degree of suspicion of COVID-19 was graded using a self-made scoring algorithm obtained by the combination of relevant symptoms, exposure to infected persons and, when available, chest computerized tomography-scan or ultrasound (Table 3). Scores ≥2 were considered suspicious and followed-up independently of test results.

**Table 3:** Clinical pre-test scoring of COVID-19 probability: a total score ≥2 is considered suspicious of infection, independently of negative tests.

**Criteria**	**Score**
Contact with COVID-19 patients	1
Fever and/or cough	2
Anosmia and/or dysgeusia	2
Dyspnea and/or shortness of breath and/or dry airways	1
Conjunctivitis and /or diarrhea and/or rash/petechia and/or muscular cramps and/or fainting/TIA/dizziness	1
HCW: >7 of 33 symptomsResidents: >3 of 18 more objectivable symptoms (see Supplementary Table 1)	1
Bilateral interstitial pneumonitis imaging (computerized tomography scan or ultrasound)	2

### RT-PCR Testing

All the 286 HCW underwent RT-PCR at the time of our integrative screening. Previous RT-PCR tests results were available in 41 workers and 146 residents (Table 4). A single nursing home (AS) had all 88 residents screened for SARS-CoV-2, as a systematic screening was performed after a hotspot of COVID-19 was detected. All remaining HCV and residents had been tested because of evocative symptoms or contact with affected people.

**Table 4:** Integrated screening interpretation: a case was considered positive for COVID-19 when 2 of 3 parameters where satisfied—a negative serology or RT-PCR test result was considered false negative only if associated with COVID-19 related symptoms or CT imaging and a positive test.

**286 HCW**	**SEROL+ CLIN+**	**SEROL– CLIN+**	**SEROL+ CLIN–**	**SEROL– CLIN–**
Previous RT-PCR positive	14	4^#^	0	0
Previous RT-PCR negative	5^*^	13	1^*^	4
Previous RT-PCR N/A	16	50^∧^	8	171
Positive repeated RT-PCR after positive serology	1^*^	/	1^*^	/
Positive repeated serology in previously RT-PCR positive discordant cases	4^#^	/	/	/
Positive serology after 60 days in RT-PCR neg cases with recent symptoms	2^∧^	/	/	/
**246 Residents**				
Previous RT-PCR positive	28	4	4	1
Previous RT-PCR negative	9	31	5^**^	64
Previous RT-PCR N/A	3	2	10	85
Positive RT-PCR repeated after positive serology	0	/	2^**^	/
False negative RT-PCR				
False negative serology				
Possible false positive serology				
Triple concordant positive SEROL/CLIN/RT-PCR				
Possible false positive RT-PCR vs. false negative serology				
Clinically suspicious tested negative				
Triple concordant negative SEROL/CLIN/RT-PCR				
Possible false positive serology vs. false negative RT-PCR				

*SEROL+: Positive serology test*.

*SEROL–: Negative serology test*.

*CLIN+: Clinical score ≥2*.

*CLIN–: Clinical score <2*.

^*^/^**^One case/two cases in common in the same column.

∧ *Two cases in common*.

# *Four cases in common*.

*RT-PCR+: positive RT-PCR test*.

*RT-PCR–: negative RT-PCR test*.

*First three lines of each group contain data of all participants at initial evaluations*.

*Following 2 HCW and 1 RESIDENTS lines contain further immediate testing in discordant cases*.

*Sixth line HCW contains serological follow up at 60 day interval*.

All RT-PCR tests were run in the same laboratory using RADI COVID-19 detection Kit (KHMedical), on ABI 7500 Bio-Rad CFX 96 platform. The lower limit of detection of this kit is 0.66 copies/microliter.

### Serology Testing

Serology screening used the rapid point-of-care test^®^ Core tests COVID-19 IgM/IgG Ab, whose instructions report IgM&IgG sensitivity 97.6% and specificity 100%. Tests reading and photographic recording were performed 15 min after deposing the finger blood sample just collected. Weak lines for IgM and IgG were considered positive, according to the report by Li et al. (12) and in consideration of the principle of maximum safety.

All those screened positive with immunoassay with no or negative RT-PCR were immediately isolated and a further swab test was taken.

Serology with the identical lot rapid Core test was repeated after 1 week in 4 HCW with discrepant negative serology screening and previous positive RT-PCR and at 60-day interval in 67 of 86 HCW with positive serology screening or with recent/suspicious clinical scores and negative tests.

Unlike HCW, residents with discrepant serology and positive virology tests did not repeat a serology test due to difficulty in getting an informed consent for further assessments. A 60-day serological follow up was also not performed.

### Statistical Analysis

Data were reported as counts and percentages. Comparison between independent proportions (groups) were performed using Fisher Exacts-test. Trends in proportions were tested using Chi-square test for trends. All analysis assumed a 5% significance level and were performed using R software (version 4.0.5).

### Informed Consent and Ethical Approval

Informed consent was obtained from all HCW and residents or their representatives.

Formal review and approval by the ethic committee of Spedali Civili of Brescia (NP 4378) was obtained for this study.

### Funding

Voluntary donations and some residual funding from previous different research studies.

## Results

A flowchart with all the results obtained is provided (Supplementary Figure 1).

### Residents

A thorough virology screening was not available for the 246 residents.

Previous RT-PCR results were available for 146 residents (59.3%—Table 1).

32/59 residents positive for serology had also been previously identified with RT-PCR while 5 cases with previous positive RT-PCR turned out negative at serology (Table 4).

Paired positive IgM and IgG bands were present in 57 of 59 seropositive cases, five of which showing weak bands. Two asymptomatic cases showed isolated IgM. No isolated IgG positivity was found.

Overall, 18/64 residents (28.1%) with positivity to either test reported complete absence of symptoms. Mild symptoms and a clinical score 0–1 were present in further 2 cases. A clinical history suspicious of COVID-19 scoring ≥2 was associated with at least one positive test in 44 residents but was also present in 31 residents with both tests negative (Table 4).

In our study the virus was rapidly spreading between residents with Alzheimer's disease or other dementias associated with wandering (30/58), while the contagion amongst all other residents (28/188) was significantly less frequent (51.7 vs. 15.9%—*p* < 0.001 Fisher exact-test). Actually, the rate of infection in residents with dementia unassociated with wandering was comparable to the group with no cognitive impairment (7/42–16.9%—*p*-value NS).

The number of infections amongst the 20 residents in persistent vegetative state was instead inferior in comparison with all other residents (1/20–5% vs. 57/226–25.2%—*p* = 0.051 Fisher exact-test).

No correlation was found between the rate of infected residents and the different size, in terms of number of residents, of these three nursing homes: 23/107 (21.5%) vs. 28/88 (31.8%) vs. 7/51 (13.7%)—*p* = 0.58 (Test for trends in proportions—*p* value NS).

### Staff

All 286 workers had SARS-CoV-2 RT-PCR, clinical and serological screening (Tables 2, 4).

Previous RT-PCR results were available for 41 HCW (14.3%).

14/44 HCW initially seropositive also had a previous positive RT-PCR result. Repeated RT-PCR in those identified with specific antibodies was positive in one additional HCW, who was thereafter isolated.

Paired positive IgM and IgG bands were present in all cases except for a single asymptomatic case showing a weak IgM band only.

Four symptomatic workers with previous positive RT-PCR and discrepant negative serology, were re-tested after 1 week with the same test-kit and all showed very weak bands for both IgM and IgG (Table 4). These four serology tests, although ultimately evaluated as positive in Table 2, have been considered false negative in Table 4, where all results are reported by chronological phases.

Follow up serology after 60 days, performed in 78% (67 of 86) of seropositive or clinically suspicious/recent HCW, yielded two additional serology-positive cases whose suspicious clinical history was recent at the time of the screening. These two additional cases have been considered serology-positive in Table 2, based on final information provided by this study.

HCW in close contact with the residents (including doctors, nurses, health care assistants, personal care-givers, physiotherapists, hairdressers, and beauticians) had significantly more infections (20.79 vs. 1.66% in remaining HCW; *p* = 0.00013 Fisher exact-test—Table 2). Familial contact with ill relatives was present in 11 HCW only, including the single administrative employee that tested positive.

The seroprevalence in the single nursing home with no COVID-related deaths amongst residents was markedly inferior (8%) to that found in the two nursing homes with known cases (19%) and appeared comparable to 7.5% seroprevalence of the general population in Lombardy at the time.^1^

The rate of positive tests was also greater amongst HCW with additional external activities in comparison to those with exclusive activity within their nursing home (20 vs. 16%—Table 2).

Among all 50 HCW with ultimately proven positivity for SARS-CoV2 (serology and/or RT-PCR) in our study, 40% only had fever and 50% reported cough (Supplementary Table 1).

At final evaluation of all test results, 9 seropositive HCW (18%) had been considered clinically non-suspicious (clinical score 0–1), 5 of which (10%) reported complete absence of symptoms. All 5 totally asymptomatic HCW had negative RT-PCR test while serology was frankly positive for both IgM and IgG in 2 cases, showed a weak IgM band only in a third case, and paired weak IgM and IgG bands in the last 2 cases. Close contact with COVID-19 patients was reported in two of them. A suspicious clinical score ≥2 was present in 41 HCW with at least one positive test but also in 61 workers who finally tested negative (Table 4).

Follow up serology after 60 days, performed in 78% (67 of 86) of seropositive or clinically suspicious/recent cases, provided identical either paired IgM and IgG or isolated weak IgM positivity patterns to those previously obtained, with no case showing disappearance of IgM or appearance of IgG. As mentioned, paired IgM and IgG bands were also demonstrated in two of the four workers with recent suspicious symptoms and negative initial serology screening.

### Isolation and RT-PCR Testing of Seropositive Cases

Viral RNA was still present in two workers and two residents re-tested after being found seropositive, three of whom were completely asymptomatic.

### Sensibility and Specificity of Clinico-Serological Screening

On the basis of the available RT-PCR ± data of participants at the time of symptoms or contacts with infected people, sensibility and specificity for on-site serology was 83 and 84%, sensibility and specificity for our clinical score was 91 and 56%, while integrated clinical scoring and on-site serology had sensibility 89% and specificity 87%.

## Discussion

Most SARS-CoV-2 diagnostic tests so far available represent “blunt weapons,” whose approval for emergency use in response to the pandemics by FDA and EC has not implied a proper estimate of performance characteristics and limitations with extensive validation tests.

### Symptoms

Symptoms of COVID-19 are poor indicators of the disease as people affected may experience a wide range of symptoms from mild to a myriad of different symptoms, and a proportion of cases between 6.3 and 96.0% (13) is asymptomatic.

Despite our scoring system intentionally devised to be highly sensitive, unexpected positive tests were still present in 9 workers and 20 residents with clinical scores below the threshold, and entirely asymptomatic disease was more frequent amongst residents (18/64–28.1% vs. 5/50–10.0%; *p* = 0.019). A similar difference has been observed elsewhere in long-term facilities (14, 15) and is most probably justified by a lesser degree of awareness and/or by difficulty in communicating with older people is particularly concerning when planning strategies for transmission containment in care homes.

In our study, the list of symptoms had been simplified since the vast majority of participants were not able to adequately report more subtle and subjective signs. This has allowed homogeneity and comparison within the same group, but it has made it impossible to assess atypical COVID-related symptoms described in older people elsewhere (16, 17). The significant paired difference in proven infection rates (64/246–26.0% vs. 50/286–17.5%; *p* = 0.020 Fisher exact-test) might be correlated with more common asymptomatic or atypical forms amongst residents.

Asymptomatic transmission is held responsible for failure of symptoms-based screenings and monitoring strategies in nursing homes (18–21). The awareness that a larger proportion of infected residents have no symptoms increases the urgency for their frequent regular testing (22).

### RT-PCR

It is considered the gold standard to identify COVID-19: however, nasal and oropharyngeal swabs are charged with a considerable quote (30–40%) of false negative results (23, 24).

In our study 7–8% of residents and workers tested at the time of symptoms/possible contact showed single or multiple false negative virology tests on nasopharyngeal swabs. Repetition of tests proved worthwhile, allowing detection of viral RNA in four cases previously testing negative.

In our study, the residents had an easier access to the virological tests in comparison to HCW. This was due to specific dispositions of our Regional Health System at the beginning of the pandemics, when the availability of tests was still not meeting the demand. In fact, access to swab testing was guaranteed to all the residents with suspicious symptoms or possible contact with an infected person after 6 weeks of the pandemics. The same option was offered to those HCW that were by profession in direct contact with the residents and directly employed by the nursing home, not including the shift workers.

### Serology

Antibody testing misses early infections but can be useful to understand the prevalence in a community, particularly when no tests have been previously available, and it may disclose false negative cases at antigen testing (25). The advent and the gradual expansion of the population vaccinated with SARS-CoV-2 vaccines, negatively impacts on the informative value provided by serology testing in the absence of associated clinical information and dynamic evaluation of antibody level changes.

### Rapid Point-Of-Care Tests

Accurate point-of-care virology and immunology tests are particularly needed for effective monitoring of an infection in vulnerable and enclosed settings (12, 26–29).

A variety of serology tests has been internationally approved since the start of COVID-19 pandemics. The diagnostic performance of quantitative serology is generally considered superior to rapid qualitative tests, but its supply has been late and is still not meeting the demand.

Point-of-care antibody tests have demonstrated efficiency comparable to that of laboratory tests (30), have been successfully used in a series of studies so far (27, 28, 31–33), and represented a valid alternative to unavailable quantitative serology in our study.

Point-of care serology tests are cheap and easy to use in number of different clinical situations (12, 26) and are particularly useful in the case of poorly cooperative older residents. Despite controversy over their accuracy (31, 34), the authors decided to follow the encouragement of WHO to use these tests in research studies to establish their usefulness. Their results require a nuanced interpretation and correlation with RT-PCR and clinical information (27, 35).

In our study, rapid on-site serology has been able to confirm symptoms in 35 persons, 20 of whom (6 HCW and 14 residents) had previous negative RT-PCR results and to unveil a previous COVID in 24 further persons with no suspicious symptoms and negative or no previous RT-PCR.

Four HCW and 5 residents had a positive RT-PCR but a negative first serology test.

Interpretation of IgM results appears particularly challenging (36). Similar to other studies of the same period (34), no case with isolated IgG was found.

Although high levels of IgM have been reported 22–24 days after symptom onset at serial serology testing (37) and several studies have shown simultaneous production of IgM and IgG against SARS-CoV-2 (12, 24, 37), the unchanged paired IgM and IgG positivity in all tests repeated after 60 days with no case with IgM disappearance is disturbing. Similarly, the two asymptomatic cases with isolated IgM and with identical positivity pattern at re-testing after 60 days raise doubts about a possible false positive result.

The combination of qualitative and quantitative antibody testing could represent a safer strategy to obtain reliable data.

Comparative evaluations of rapid tests for COVID-19 infection in care homes are urgently needed too (28).

### Identifying Weaknesses

Similar to other studies (7, 38), serology allowed a retrospective evaluation of the control strategies adopted. Besides continuous attention to maintain adherence to the measures put in place and regular testing, identifying weaknesses is crucial to safety in nursing homes.

Laboratory testing capacity was lagging for several weeks after the COVID-19 outbreak in Italy (6). While our regional government focused on hospitals and locating available ICU beds, Lombardy's nursing homes were in many ways left to fend for themselves with no access to RT-PCR testing for weeks. This has negatively interfered with the ability to contain the spread of disease.

The size of a care home is related to the probability of viral contamination, in relation to the number of residents and staff and the presence/ absence of shift workers. This seems confirmed by our results, but the small number of care homes studied does not allow significant conclusion.

The structure of the building itself and the proportion of available single rooms are determinant factors in containment of infection. Although the data of our three nursing homes do not provide confirmation of this hypothesis, a greater representative sample size would be needed to assess it.

The high rate of infected staff in close contact with residents is probably linked to lack of using PPE during the initial days of the outbreak, when no cases had yet been identified or no PPE was available.

Tracing contacts and clarifying the chain of viral transmission in the confined crowded environment of a nursing home might be difficult (35), especially in the absence of laboratory testing capacity. The quick viral spread observed amongst residents with wandering dementia, should be considered when planning strategies to contain the spread of the disease. Moreover, the scientific community has been questioning the ethical implications of implementing testing and restrictive measures such as isolation for residents with dementia. These people are unable to understand the significance of such measures that deeply modify their daily routine (39–41). It is well-documented that reduced social interactions have a greater impact on the residual capacities of these subjects compared to the general population. Therefore, future guidelines relating to restrictive measures must also take this into consideration (22).

Our study highlights the difficulty to gain access to RT-PCR for SARS-CoV-2 infection at the beginning of the pandemics in Italy, the potential contribution of clinical score associated with RT-PCR for SARS-CoV-2 infection diagnosis, and the usefulness of rapid serology tests to assess the level of the COVID-19 outbreak diffusion in nursing homes.

Our data show that a combined strategy with nasopharyngeal swabs, rapid on-site serology and accurate anamnestic monitoring has contributed to reveal the real diffusion of SARS-CoV-2. Our approach has increased the awareness of a previous infection in people that could not get a virology test or had a false negative in the past, clarifying the chain of transmission and identifying symptomatic and asymptomatic persons actually carrying the virus.

The contribution of rapid serology tests in unveiling previous infections is now less in the more developed countries where vaccines have been already administered to the people in nursing and care homes. However, our proposed triple screening strategy could still be a valuable option for daily practice in countries with reduced or no national health service. It could also guide the prioritization of the more vulnerable residents for access to vaccines or the decision to omit the second dose in case of a previous documented immunization (42).

However, continuous monitoring of symptoms and implementation of protective measures is mandatory given the frequent appearance of new variants of SARS-CoV-2 worldwide that might escape the protection provided by the vaccines currently available.

## Conclusions

Although escaping infection so far, vulnerable residents and staff of nursing homes are still at risk for COVID-19, especially in countries where the availability of laboratory testing is not meeting the demand of a regular monitoring strategy and vaccines are not yet available.

Our data show that an integrated approach with continuous monitoring of symptoms, coupled with periodic rapid on-site antigen and antibody testing of residents and staff could be part of a strategy surveillance system to increase protection. This is particularly relevant where laboratory testing for SARS-CoV-2 is not available or not in keeping with the international recommendations for timely identification and control of outbreaks^2^.

Additional studies addressing the performance of point-of-care tests in long-term facilities are urgently needed.

## Data Availability Statement

The raw data supporting the conclusions of this article will be made available by the authors, without undue reservation.

## Ethics Statement

This study was reviewed and approved by the Ethics Committee of Spedali Civili of Brescia (NP 4378). The patients/participants or their relatives provided their written informed consent to participate in this research.

## Author Contributions

AS and EM prepared the manuscript, performed rapid serological tests, clinical assessment, and analyzed the results. SC performed the statistical analysis. GG and VR provided medical care, clinical information of residents, supplied RT-PCR test results of residents, and staff included in the study. All authors contributed feedback, revisions of the manuscript draft, read, and approved the final manuscript.

## Conflict of Interest

The authors declare that the research was conducted in the absence of any commercial or financial relationships that could be construed as a potential conflict of interest.

## Footnotes

^1^https://www.istat.it/it/files//2020/08/ReportPrimiRisultatiIndagineSiero.pdf (accessed on August 4, 2020).

^2^https://www.ecdc.europa.eu/sites/default/files/documents/Infection-prevention-and-control-in-healthcare-settings-COVID-19_5th_update.pdf (accessed on May 5, 2021).

## Supplementary Material

Supplementary Figure 1 Flowchart of triple screening study. RES, Residents; HCW, Health Care Workers; SEROL, Serology (+ positive; − negative); clin neg, Below the threshold of clinical assessment (score 0–1); PREV RT-PCR, Previous RT-PCR (+ positive; − negative; N/A Not Available).

Supplementary Table 1 List of symptoms and their prevalence in 50 HCW and 64 residents with proven positivity for SARS-CoV2 (PCR and/or serology positive).
