# Supplementary figures and images for: Rapid Point-Of-Care Serology and Clinical History Assessment Increase Protection Provided by RT-PCR Screening: A Pilot Study Involving Three Nursing Homes in Brescia, a Hotspot of Lombardy
Source: Front Public Health. 2021 Jun 24;9:649524. doi: 10.3389/fpubh.2021.649524 (PMC8264443; doi:10.3389/fpubh.2021.649524)

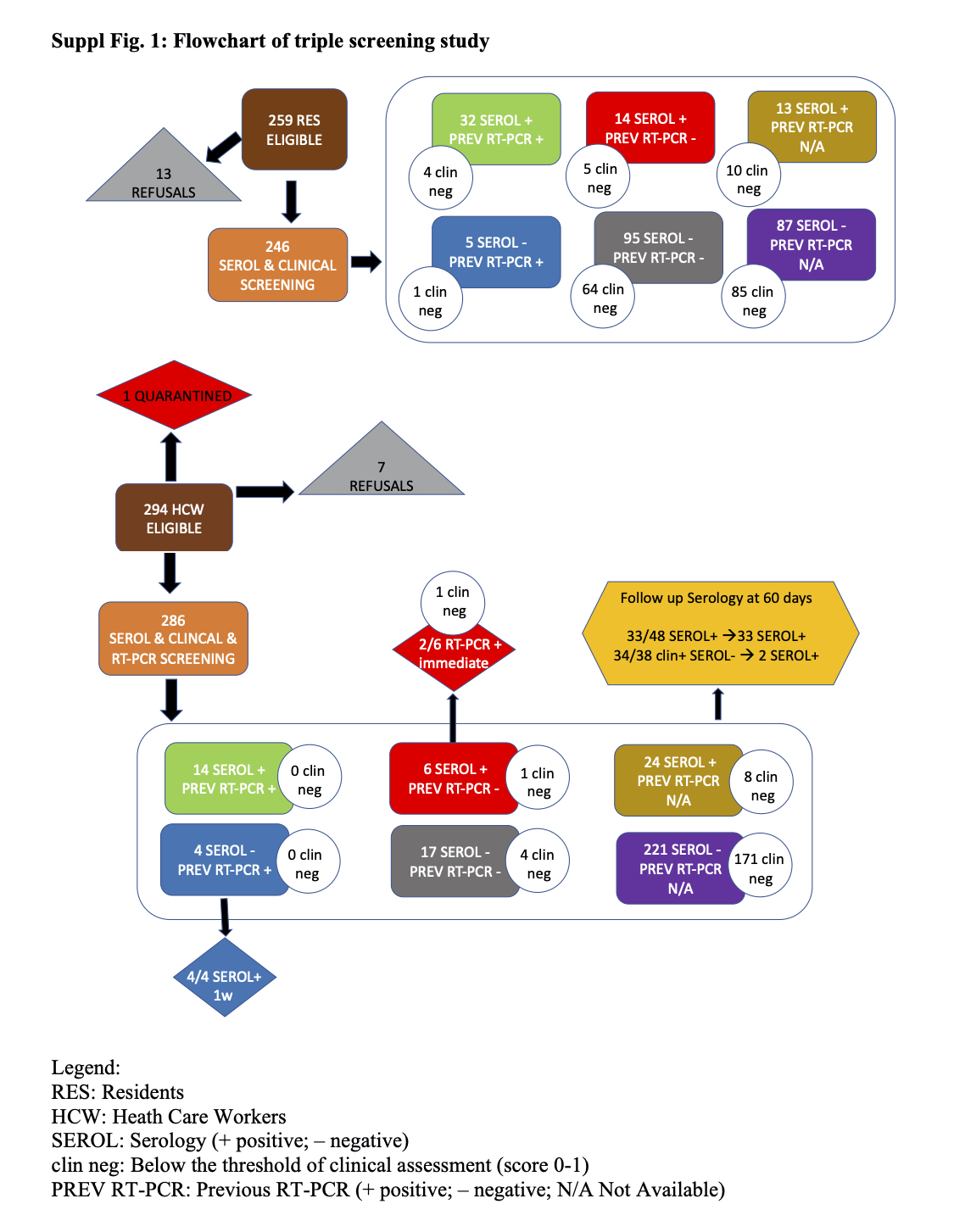

Supplement: Supplementary Figure 1 — Flowchart of triple screening study. RES, Residents; HCW, Health Care Workers; SEROL, Serology (+ positive; − negative); clin neg, Below the threshold of clinical assessment (score 0–1); PREV RT-PCR, Previous RT-PCR (+ positive; − negative; N/A Not Available). [file Image_2.JPEG]

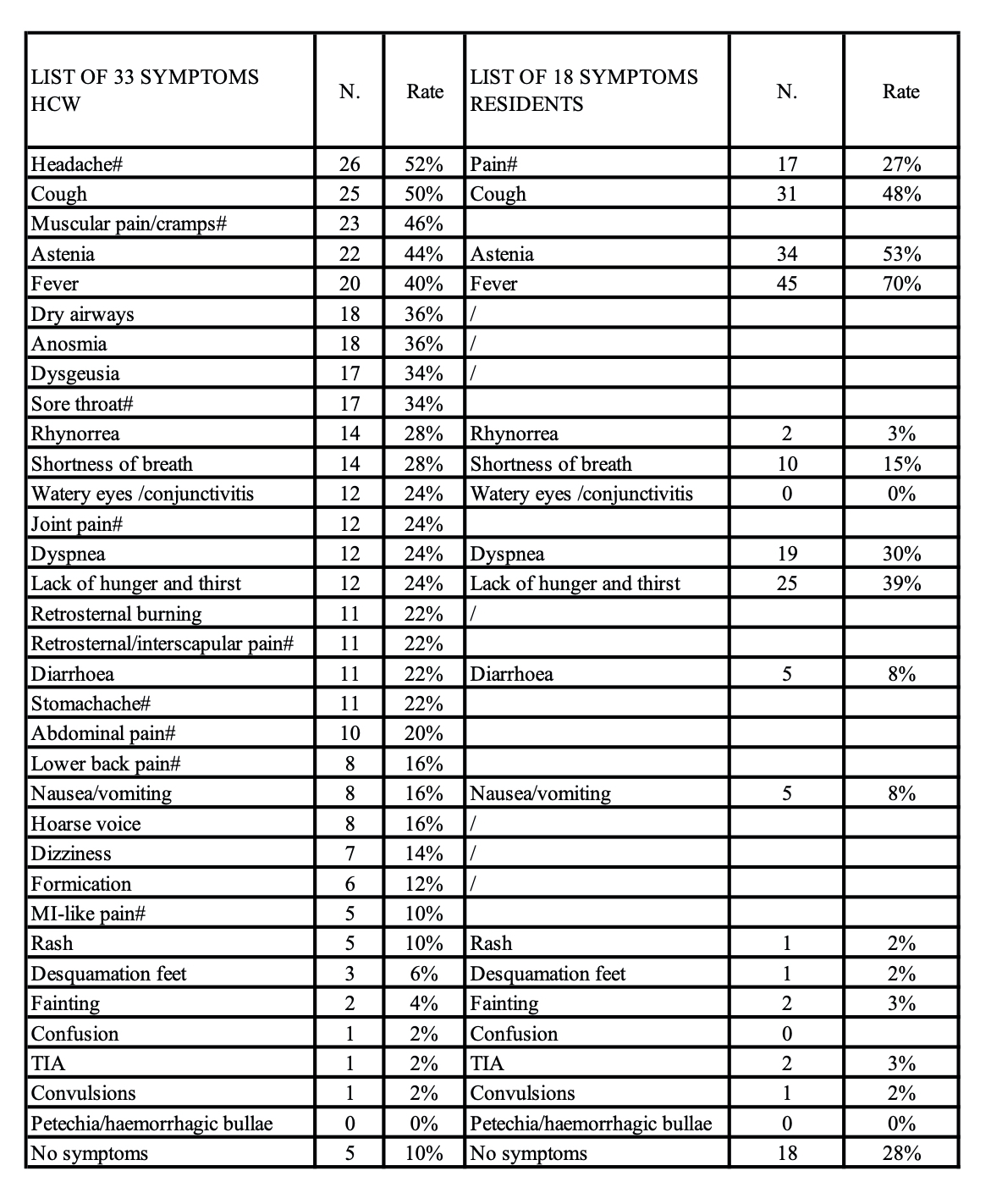

Supplement: Supplementary Table 1 — List of symptoms and their prevalence in 50 HCW and 64 residents with proven positivity for SARS-CoV2 (PCR and/or serology positive). [file Image_1.JPEG]
